# Type 1 Interferon Gene Signature Promotes RBC Alloimmunization in a Lupus Mouse Model

**DOI:** 10.3389/fimmu.2020.584254

**Published:** 2020-09-25

**Authors:** June Young Lee, Emaan Madany, Najwa El Kadi, Sumaarg Pandya, Kessandra Ng, Michifumi Yamashita, Caroline A. Jefferies, David R. Gibb

**Affiliations:** ^1^Department of Pathology and Laboratory Medicine, Cedars-Sinai Medical Center, Los Angeles, CA, United States; ^2^Department of Internal Medicine, Division of Rheumatology, Cedars-Sinai Medical Center, Los Angeles, CA, United States; ^3^Division of Transfusion Medicine, Cedars-Sinai Medical Center, Los Angeles, CA, United States

**Keywords:** RBC alloimmunization, type 1 interferons, lupus, transfusion, autoimmunity

## Abstract

Red blood cell (RBC) transfusion exposes recipients to hundreds of unmatched minor RBC antigens. This exposure can lead to production of alloantibodies that promote clinically significant hemolytic events. Multiple studies have reported an increased frequency of RBC alloimmunization in patients with autoimmunity. However, cellular and molecular mechanisms that underlie autoimmunity-induced alloimmunization have not been reported. Patients with systemic lupus erythematosus (SLE) have a high frequency of alloimmunization and express a type 1 interferon (IFNα/β) gene signature. Thus, we utilized the pristane-induced lupus mouse model to test the hypothesis that inflammation in lupus promotes RBC alloimmunization, and to examine the potential role of IFNα/β. Intraperitoneal injection of pristane, a hydrocarbon oil, led to autoantibody production, glomerulonephritis, and pulmonary hemorrhage in wild type (WT) mice. Pristane treatment significantly induced serum IFNα and expression of multiple interferon-stimulated genes (ISGs) in peripheral blood and peritoneal fluid cells, including inflammatory macrophages. Following transfusion with allogeneic RBCs expressing the KEL glycoprotein, pristane-treated WT mice produced significantly elevated levels of anti-KEL IgM and anti-KEL IgG, compared to untreated mice. Pristane induced comparable levels of inflammatory cells and cytokines in mice lacking the IFNα/β receptor (IFNAR1^–/–^) or the IFNα/β-inducing transcriptions factors (IRF3/7^–/–^), compared to WT mice. However, pristane-treated IFNAR1^–/–^ and IRF3/7^–/–^ mice failed to produce ISGs and produced significantly lower levels of transfusion-induced anti-KEL IgG, compared to WT mice. Thus, pristane induction of a lupus-like phenotype promoted alloimmunization to the KEL RBC antigen in an IFNα/β-dependent manner. To our knowledge, this is the first examination of molecular mechanisms contributing to RBC alloimmunization in a model of autoimmunity. These results warrant further investigation of the role of IFNα/β in alloimmunization to other RBC antigens and the contribution of the IFNα/β gene signature to the elevated frequency of alloimmunization in patients with SLE.

## Introduction

Red blood cell (RBC) transfusion exposes recipients to hundreds of non-ABO RBC antigens that are not routinely matched between donors and recipients. This exposure can lead to production of anti-RBC alloantibodies that promote clinically significant hemolytic events, including potentially fatal hemolytic transfusion reactions, hemolytic disease of the newborn, and rejection of renal allografts expressing allo-antigens also expressed on RBCs (1–4). Additionally, RBC alloimmunization against multiple RBC antigens can prohibit the availability of compatible RBC units, leading to anemia-associated morbidity and mortality (5, 6). Identifying factors that promote RBC alloantibody responses would allow for identification of at-risk patients who may benefit from interventions that inhibit alloimmunization.

One such factor is the state of inflammation in the transfused recipient. In 1995, Ramsey and Smietana reported that women with autoimmune disease have a high incidence of RBC alloimmunization (7). Subsequent studies showed increased alloimmunization in patients with various autoimmune diseases, including inflammatory bowel disease, rheumatoid arthritis, autoimmune hepatitis, and systemic lupus erythematosus (SLE) (8–10). For example, while 3–5% of all transfused patients form RBC alloantibodies, (11, 12) more than 20% of transfused patients with SLE produce such alloantibodies, representing the second highest incidence, compared to other studied disease populations (8). However, the cellular and molecular mechanisms underlying the elevated incidence in patients with SLE or other chronic autoimmune diseases, including the role of inflammatory pathways, have not been investigated.

A pathway shown to promote autoantibody production and disease severity in SLE is type 1 interferon (IFNα/β) production and signaling. While IFNα/β was discovered for its critical role in anti-viral immunity (13), more recent studies have implicated IFNα/β in the pathogenesis of autoimmune diseases, including rheumatoid arthritis, myositis, Sjögren’s syndrome (SS), systemic sclerosis and SLE (14–18). Approximately two-thirds of adult patients, and nearly all children, with SLE express an IFNα/β gene signature, defined as the expression of multiple interferon-stimulated genes (ISGs) (19–23). In addition, more than 50% of SLE-associated genetic variants have been linked to the IFNα/β pathway (24).

While the contribution of an autoimmune gene signature to RBC alloimmunization has not been examined in humans or animal models, treatment of murine transfusion recipients with specific inflammatory stimuli has been shown to induce or enhance RBC alloimmune responses (11, 25, 26). Co-transfusion with CpG DNA or pre-treatment with polyinosinic:polycytidylic acid [poly(I:C)], a mimetic of viral double stranded RNA, has been shown to induce alloimmunization to a human RBC antigen expressed on mouse RBCs (25–27). Accordingly, polyoma and influenza viral infections in mice enhance the alloimmune response to transfused RBCs (28, 29). Prior studies have also shown that transfusion of pro-inflammatory stored RBCs induces alloimmunization (30–32). Arneja et al. demonstrated that IL-6 is critical for T follicular helper cell promotion of RBC alloimmune responses to stored RBCs (33). Finally, it is noteworthy that not all inflammatory stimuli promote alloimmunization, as gram-negative bacteria and lipopolysaccharide have been shown to inhibit alloimmunization (34, 35). Thus, activation of specific inflammatory pathways in certain conditions can promote or inhibit RBC alloimmunization.

Compared to viral infection and acute stimuli, inflammatory pathways in SLE and other autoimmune diseases differ in strength and chronicity. In patients with chronic autoimmunity, the role of IFNα/β and other cytokine-mediated inflammatory pathways in RBC alloantibody responses is poorly understood. Here, given the increased frequency of alloimmunization in patients with SLE, we examine the degree to which inflammation in a lupus mouse model influences RBC alloimmunization. Use of the pristane-induced lupus model, which exhibits a strong IFNα/β signature (36), also allows examination of the role of IFNα/β in alloimmunization in an autoimmune model.

## Materials and Methods

### Mice

C57BL/6 and IFNAR1^–/–^ mice were purchased from Jackson Laboratories (Bar Harbor, ME, United States). IFNAR1^–/–^, IRF3^–/–^, IRF7^–/–^, and K1 RBC transgenic mice were previously described (37–39). K1 mice express the KEL glycoprotein, containing the KEL1 antigen, on RBCs. Appropriate gene-deficient mice were bred to produce IRF3/7^–/–^ double knockout mice. All mice were 8–12 weeks of age and had been backcrossed to the C57BL/6 background for more than 8 generations. All pristane-treated mice were female mice injected intraperitoneally with one dose of 0.5 mL pristane (2,6,10,14-tetramethylpentadecane). All animal protocols were approved by the Cedars-Sinai Institutional Animal Care and Use Committee.

### RBC Transfusion

Peripheral blood of K1 mice was collected in 12% Citrate Phosphate Dextrose Adenine (CPDA-1, Jorgensen Labs, Melville, NY, United States) by retro-orbital bleeding and leuko-reduced with a Pall (East Hills, NY, United States) syringe filter. Recipient mice were transfused i.v. with 75 μL of leuko-reduced packed RBCs, which is the approximate mouse equivalent of 1 unit of human RBCs.

### Clearance Assay

For post-transfusion recovery experiments, mice previously transfused with K1 RBCs received a second transfusion of fluorescently labeled K1 and C57BL/6 RBCs 35 days after the first transfusion. K1 and C57BL/6 RBCs were labeled with DiI (1,1’-Dioctadecyl-3,3,3’,3’-Tetramethylindocarbocyanine Perchlorate) and DiO (3,3’-Dioctadecyloxacarbocyanine Perchlorate) lipophilic dyes (Life Technologies, Camarillo, CA, United States), respectively, according to manufacturer’s instructions. A 2:1 ratio of K1 to C57BL/6 RBCs was mixed and transfused into previously transfused recipients and K1 transgenic mice, which served as a negative control. Mice were bled 10 min, 1, 2, and 4 days after transfusion, and the ratio of K1 to C57BL/6 RBCs remaining in circulation was measured by flow cytometry. The percentage of K1 RBCs remaining in circulation, compared to C57BL/6 RBCs remaining, was plotted as post-transfusion recovery.

### Measurement of Anti-KEL Alloantibodies

Serum anti-KEL IgM, IgG, IgG1, IgG2b, IgG2c, and IgG3 were measured by flow cytometric crossmatch. Anti-KEL IgM was measured 5 days after transfusion, and anti-KEL IgG was measured 7, 14, 21, and 28 days after transfusion as previously described (40). Briefly, serum from transfused mice was incubated with K1 or C57BL/6 RBCs and subsequently stained for RBC-bound IgM or IgG (goat anti-mouse IgM FITC or goat anti-mouse IgG APC). Secondary antibodies for IgG subsets included goat anti-mouse IgG1 PE, goat anti-mouse IgG2c APC, goat anti-mouse IgG2b FITC, and goat-anti mouse IgG3 BV421 (Jackson ImmunoResearch, West Grove, PA, United States). The adjusted MFI was calculated by subtracting the reactivity of serum with syngeneic C57BL/6 RBCs from the reactivity of serum with K1 RBCs. Graphed anti-KEL IgG data represents the peak antibody response, 21–28 days following transfusion. Flow cytometry of RBCs was performed using a SONY SA 3800 (San Jose, CA, United States) or a Cytek Northern Lights 3000 spectral analyzer (Fremont, CA, United States) and analyzed using FlowJo software (Tree Star, Ashland, OR, United States).

### Flow Cytometric Analysis of Leukocytes

Single cell suspensions of peripheral blood leukocytes and splenocytes were analyzed following RBC lysis. Peritoneal fluid cells were collected by flushing the peritoneal cavity with media. Spleens were minced with a razor blade prior to cell filtration with a 70 μM nylon mesh. Suspensions were incubated with Fc receptor block, TruStain FcX, from Biolegend (San Diego, CA, United States) and stained with fluorescently conjugated antibodies specific for cell surface markers, including CD11c (clone N418), B220 (RA3-6B2), F4/80 (BM8), CD11b (M1/70), Ly6C (HK1.4), Ly6G (1A8), Siglec-1 (3D6.112), TCRβ (H57-597), and I-A/I-E (MHC II, M5/114.15.2) from Biolegend. Zombie-Red and Zombie-NIR (Biolegend) were used to exclude dead cells. Cells were acquired with a Cytek Northern Lights 3000 or a LSRII flow cytometer (Becton Dickinson, San Jose, CA, United States) and analyzed using FlowJo.

### Quantitative PCR

RNA was isolated from peritoneal fluid and peripheral blood leukocytes using the Qiagen RNeasy mini-kit (Hilden, Germany) and converted to cDNA with the Maxima H Minus cDNA Synthesis Master (Thermo Fisher Scientific, Waltham, MA, United States). The amount of GADPH, Mx1, ISG15, and IRF7 cDNA was measured by a QuantStudio 5 Real-Time PCR System using PowerUp SYBR Green master mix (Thermo Fisher Scientific). Primer sequences are listed in Supplementary Table 1. Thermo Fisher Scientific Connect software was used to determine the relative expression of target genes, compared to GAPDH.

### ELISAs, Creatinine, and Hematocrit

Serum autoantibodies and urine albumin were measured by ELISA using the mouse anti-SSA (RO-52) ELISA kit (Signosis, Inc., Santa Clara, CA, United States), the mouse anti-dsDNA IgG ELISA Kit (Alpha Diagnostic International, San Antonio, TX, United States), and the Albuwell M ELISA kit (Ethos Biosciences Inc., Philadelphia, PA, United States). Urine creatinine was measured using a Creatinine Liquicolor kit (Stanbio Laboratory, Boerne, TX, United States). Hematocrit was measured using the HemaTrue^®^ Veterinary Hematology Analyzer (Heska, Loveland, CA, United States).

### Histology

Lungs and kidneys were fixed in 10% buffered formalin (Medical Chemical Corporation, Torrance, CA, United States). Slides were cut from paraffin-embedded blocks by the Cedars-Sinai histology lab and stained with hematoxylin and eosin for lungs and periodic acid-Schiff for kidneys. Lung and renal histology slides were scored by a renal pathologist (M.Y.).

### Cytokine Measurement

Serum was collected by retro-orbital bleeding and centrifugation, and cytokine measurement and analysis were performed with the LEGENDplex mouse anti-virus response panel multiplex assay (Biolegend) according to manufacturer’s instructions.

### Statistics

Statistical analysis was performed using Graph Pad Prism software (San Diego, CA, United States). Statistical significance between two groups was determined using an unpaired Student’s *t*-test or Mann Whitney *U* test for parametric and non-parametric data, respectively. Significance between three groups of non-parametric data was determined using a Kruskal-Wallis test with a Dunn’s post-test. Anti-KEL antibody levels and cytokine data are non-parametric. Data bars represent the mean. Error bars represent the standard error of the mean. White circles indicate data from individual mice.

## Results

### Pristane-Induced Autoimmune Pathology

We utilized the previously described pristane-induced lupus model (41) to assess the effect of lupus-like inflammation on RBC alloantibody responses. Pristane is a hydrocarbon oil that, when injected intraperitoneally, leads to a lupus-like phenotype by a toll-like receptor 7 (TLR7)-dependent mechanism (42). In accordance with prior studies using C57BL/6 mice, the mortality caused by pristane treatment was 0–20% for all experiments (data not shown) (43). Compared to untreated wild type (WT) mice, pristane-treated WT mice produced elevated levels of lupus-associated autoantibodies, including anti-Sjögren’s syndrome related antigen A (SSA) and anti-dsDNA IgG (Supplementary Figures 1A,B). Eight months following pristane treatment of WT C57BL/6 mice, glomeruli exhibited mild mesangial expansion and hypercellularity (Supplementary Figures 1C,D), as previously reported (44). However, pristane-induced glomerular changes did not consistently lead to a significant increase in the urine albumin:creatinine ratio, compared to untreated WT mice (Supplementary Figure 1E). Also consistent with prior studies in C57BL/6 WT mice, pristane-treated mice developed diffuse pulmonary hemorrhage (Supplementary Figures 1F–H) and anemia (Supplementary Figure 1I) two weeks following pristane treatment (44, 45).

We examined the degree to which pristane induced anti-RBC autoantibodies by performing direct antiglobulin tests (DATs), which detect RBC-bound IgG. Two weeks after pristane treatment, none of the WT mice had positive DATs (data not shown). After 8 months, the results were highly variable. On occasion, pristane-treated WT mice had positive DATs (Supplementary Figure 2A), but collectively, there was no significant difference in the percent of RBCs with bound IgG, between pristane-treated and untreated WT mice (Supplementary Figure 2B).

### Induction of Anti-KEL Alloimmunization in Pristane-Induced Lupus Mice

**FIGURE 1:**
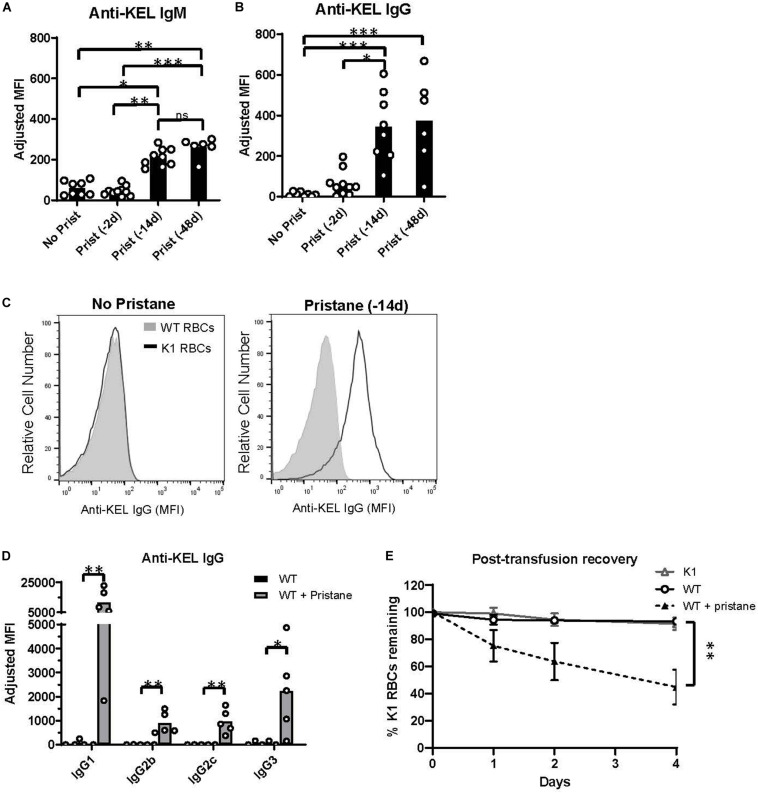
Pristane induces anti-KEL alloantibodies. Recipient WT mice were transfused with K1 RBCs. **(A,B)** Serum anti-KEL IgM and IgG in untreated mice and mice treated with pristane 2, 14, or 48 days prior to transfusion, measured by flow cytometric crossmatch. The adjusted MFI was calculated by subtracting the MFI of serum incubated with WT RBCs from the MFI of serum incubated with K1 RBCs, shown in **(C)**. **(C)** Representative histograms of flow cytometric crossmatch, in which post-transfusion serum was incubated with K1 or WT RBCs. **(D)** Anti-KEL IgG subtypes in mice treated with or without pristane 14 days prior to transfusion. **(A–D)** Anti-KEL IgM was measured 4–5 days after transfusion. Anti-KEL IgG and subtypes represent the peak IgG response 21–28 days after transfusion. **(E)** WT mice treated with or without pristane 14 days prior to transfusion were re-transfused with a 2:1 mixture of labeled K1 and WT RBCs 35 days after the first transfusion. Control K1 recipients are negative controls. Ratios of recovered K1 and WT RBCs are calculated as percent of K1 RBCs remaining 1, 2, and 4 days after transfusion. **(A–E)** Representative of 3 independent experiments, 5–10 mice per group. *p < 0.05, **p < 0.01, ***p < 0.001 by Mann-Whitney *U* test **(D)** or Kruskal-Wallis test with a Dunn’s post-test **(A,B,E)**.

To examine RBC alloimmunization in pristane-induced lupus mice, we utilized the previously described mouse transfusion model, in which RBCs from transgenic mice expressing the KEL glycoprotein (K1 RBCs) are transfused into allogeneic recipients (37). Leuko-reduced K1 RBCs were transfused into WT mice treated without or with pristane 2, 14, or 48 days prior to transfusion, and anti-KEL IgM and IgG antibody levels were measured by flow cytometric crossmatch. Consistent with prior studies (29, 37), transfused untreated mice produced low levels of anti-KEL IgM, and produced nearly undetectable levels of anti-KEL IgG. Transfusion 2 days after pristane treatment did not induce elevated alloantibody production, compared to untreated mice. However, recipients treated 14 or 48 days prior to transfusion produced significantly higher levels of anti-KEL IgM and anti-KEL IgG, compared to untreated WT mice (Figures 1A–C). There was no significant difference in anti-KEL IgG produced in mice treated 14 or 48 days before transfusion (Figure 1B and Supplementary Figure 3). Thus, further analysis was completed in mice treated with pristane 14 days prior to transfusion, unless otherwise specified. Anti-KEL IgG in pristane-induced lupus mice included all IgG subclasses made in C57BL/6 mice (Figure 1D).

To test the clinical significance of anti-KEL antibodies in pristane-induced lupus mice, we examined the ability of anti-KEL antibodies to clear K1 RBCs from circulation. Previously transfused pristane-treated and untreated mice received a second transfusion of a mixture of fluorescently labeled K1 RBCs (DiI+) and WT RBCs (DiO+) 35 days after the first transfusion. K1 mice were also transfused to serve as a negative control. The ratio of K1 RBCs to WT RBCs remaining in circulation was measured by flow cytometry. Compared to K1 and untreated WT recipients, pristane-treated mice preferentially cleared K1 RBCS within 4 days following the second transfusion (Figure 1E).

### Pristane-Induced Inflammation in the Peri-Transfusion Period

**FIGURE 2:**
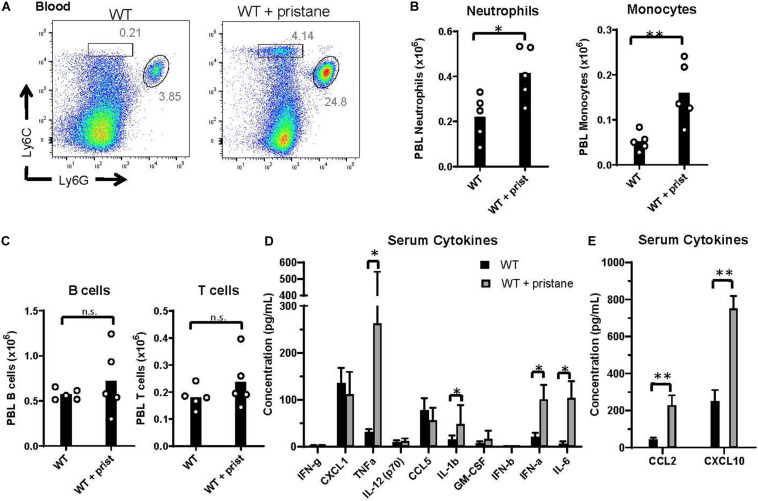
Pristane-induced inflammation in peripheral blood. WT mice were treated with or without pristane 14 days prior to analysis. **(A)** Representative flow cytometric analysis of Ly6C^+^ monocytes and Ly6G^+^ neutrophils gated on live cells. Numbers on plots indicate percentage within drawn gates. **(B)** Quantification of neutrophils and monocytes gated in **(A)**. **(C)** Quantification of B220^+^ B cells and TCRβ^+^ T cells. **(A–C)** Cells are gated on Zombie negative live cells. **(D,E)** Serum cytokine levels measured by multiplex array. Representative of 3 independent experiments with 5 mice per group. *p < 0.05, **p < 0.01, by student’s *t*-test **(B,C)** or Mann-Whitney *U* test **(D,E)**.

Prior studies using K1 mice indicate that inflammation at the time of transfusion influences alloimmunization (29, 37). Thus, given the elevated alloantibody responses and clearance in pristane-induced lupus mice, we examined markers of inflammation at the time of transfusion. While there were no significant differences in the number of B and T cells between groups, Ly6G^+^ neutrophils and Ly6G^–^ Ly6C^+^ monocytes were significantly elevated in the peripheral blood of pristane-treated WT mice (Figures 2A–C). Additionally, levels of spleen neutrophils, monocytes, CD11b^+^ F4/80^+^ macrophages, and CD11c^hi^ MHCII^+^ conventional dendritic cells were elevated in pristane-treated mice (Supplementary Figure 4).

Serum cytokine analysis showed that TNFα, IL-1b, IL-6, and IFNα were significantly elevated in pristane-treated mice, compared to untreated mice, in 3 of 3 experiments (Figure 2D). IFNβ was significantly elevated in 1 of 3 experiments (data not shown). In addition, the interferon stimulated genes (ISGs), CCL2 and CXCL10, were elevated in serum of pristane-treated mice (Figure 2E). To determine the chronicity of pristane-induced cytokine production, we quantified serum cytokine and ISG levels in mice treated with or without pristane 2 days, 14 days, or 8 months prior to analysis. Two days after pristane treatment, TNFα, IL-1b, IL-6, IFNα, CCL2, and CXCL10 were not elevated compared to untreated mice. In addition, there was no significant difference in cytokine or ISG levels between mice treated 14 days or 8 months prior (Supplementary Figure 5). This indicates that cytokines and ISGs are chronically produced from 2 weeks to 8 months after pristane treatment. It is also notable that cytokine and ISG production significantly elevated 14 days, but not 2 days, after pristane treatment coincided with transfusion-induced anti-KEL IgM and IgG production.

**FIGURE 3:**
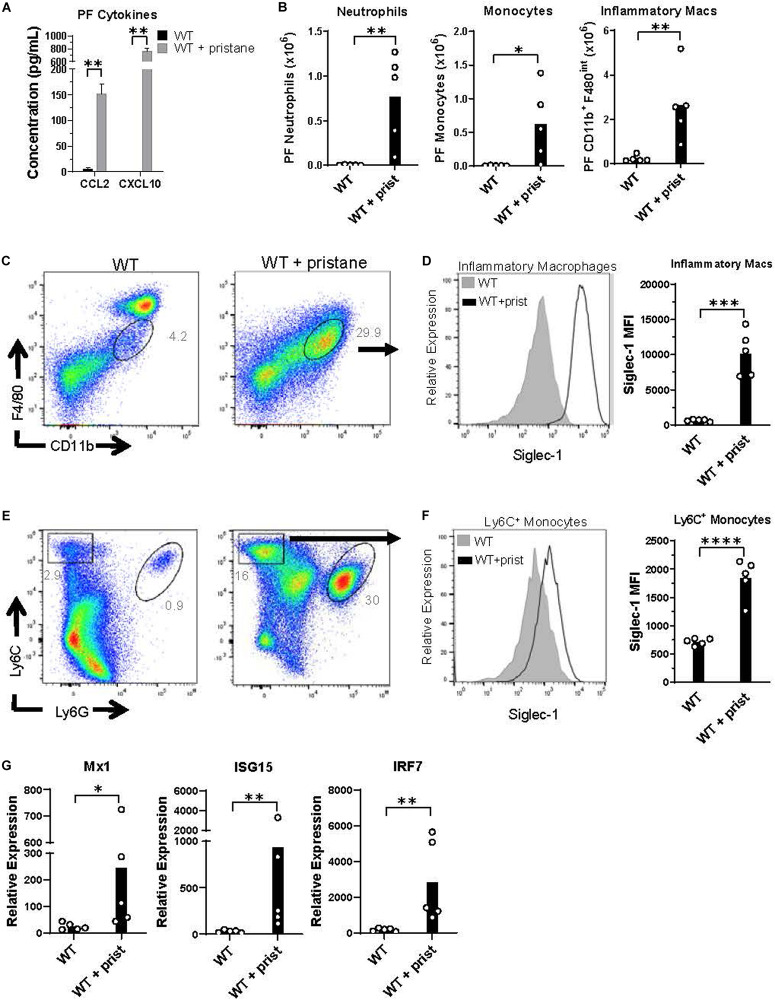
Inflammatory cells in peritoneal fluid express ISGs at the time of transfusion. WT mice were treated with or without pristane 14 days prior to analysis. **(A)** CCL2 and CXCL10 levels in peritoneal lavage fluid measured by multiplex array. **(B)** Quantification of peritoneal fluid neutrophils, monocytes, and inflammatory macrophages gated in **(C,E)**. **(C,E)** Representative flow cytometric analysis of CD11b^+^F4/80^*int*^ inflammatory macrophages (gated on live non-lymphocytes, Zombie^–^ TCRβ^–^ B220^–^), Ly6C^+^ monocytes and Ly6G^+^ neutrophils in peritoneal fluid, gated on Zombie negative live cells. **(D,F)** Representative histograms (left) and quantification (right) of Siglec-1 expression by CD11b^+^F4/80^*int*^ inflammatory macrophages and Ly6C^+^ monocytes gated in **(C,E)**. **(G)** Relative expression of Mx1, ISG15, and IRF7, compared to GAPDH, by all peritoneal fluid cells, measured by quantitative real-time PCR. Representative of 3 independent experiments. *p < 0.05, **p < 0.01, ***p < 0.001, ****p < 0.0001 by student’s *t*-test **(B,D,F)** or Mann-Whitney *U* test **(A,G)**.

Prior studies have shown that pristane induces chronic inflammation in the peritoneum (41). Thus, peritoneal fluid was evaluated at the time of transfusion, and CCL2 and CXCL10 were found to be significantly elevated in pristane-treated mice (Figure 3A). CCL2 and CXCL10 are chemokines that recruit inflammatory macrophages, monocytes, and neutrophils to sites of inflammation. These cell subsets were elevated in the peritoneal fluid of pristane-treated mice (Figure 3B). Consistent with other models of peritoneal inflammation (46), pristane treatment led to a reduction of CD11b^+^ F4/80^hi^ resident macrophages, present in untreated mice, and an increase in inflammatory CD11b^+^ F4/80^*int*^ inflammatory macrophages (Figure 3C). Given the elevated levels of IFNα and ISGs in peripheral blood, ISG expression was measured in peritoneal fluid cells. Siglec-1 was previously shown to be upregulated on monocytes following IFNα/β signaling (47). Accordingly, Ly6C^+^ monocytes and CD11b^+^ F4/80^*int*^ inflammatory macrophages expressed elevated levels of Siglec-1 (Figures 3C–F). In addition, collectively, peritoneal fluid cells expressed elevated levels of other ISGs, including Mx1, ISG15, and TLR7 compared to untreated mice (Figure 3G).

### IFNα/β-Independent Inflammatory Cell and Cytokine Responses

**FIGURE 4:**
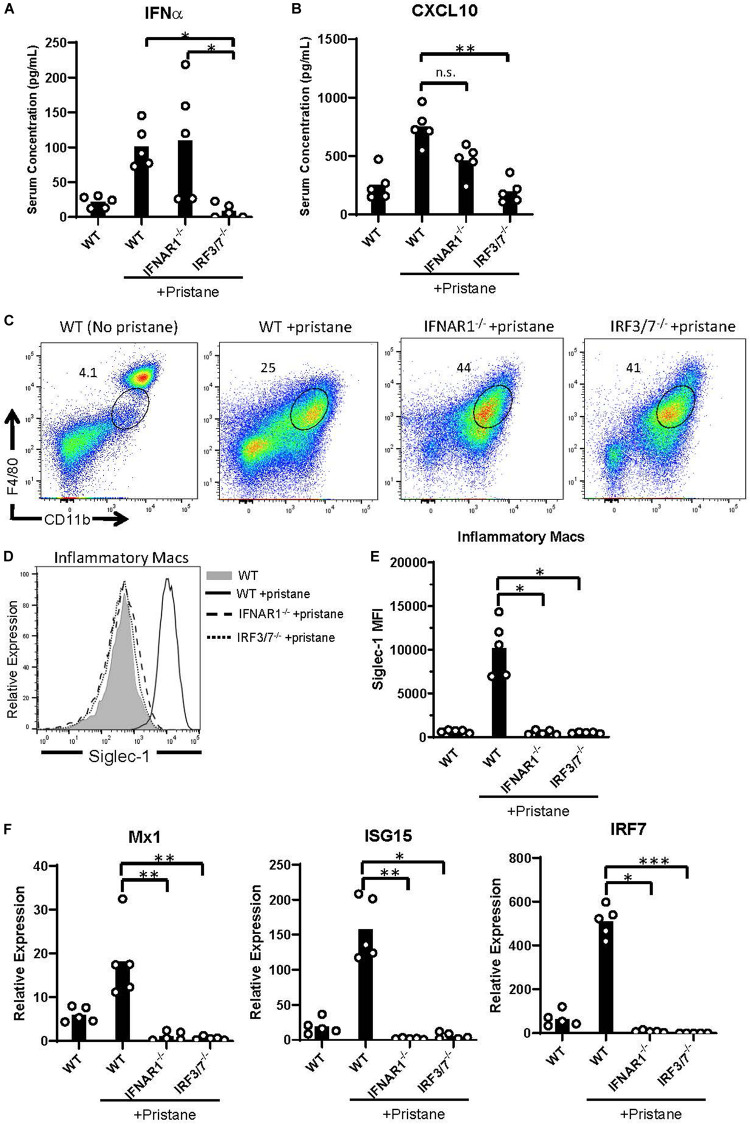
Abrogated ISG expression in pristane-treated IFNAR1^– /–^ and IRF3/7^– /–^ mice. WT, IFNAR1^– /–^, and IRF3/7^– /–^ mice were treated with pristane 14 days prior to analysis. Untreated WT mice were included as controls. **(A,B)** Serum IFNα and CXCL10 levels measured by multiplex array. **(C)** Representative flow cytometric plots of CD11b^+^ F4/80^*int*^ inflammatory macrophages, gated on live non-lymphocytes (Zombie^–^ TCRβ^–^ B220^–^) cells. Numbers on plots indicate percent of cells within drawn gates. **(D)** Histogram overlays and **(E)** quantification of Siglec-1 expression by inflammatory macrophages gated in **(C)**. **(F)** Relative expression of Mx1, ISG15, and IRF7, compared to GAPDH, by all peritoneal fluid cells, measured by quantitative real-time PCR. Representative of 3 independent experiments. *p < 0.05, **p < 0.01, ***p < 0.001 by Kruskal-Wallis test with a Dunn’s post-test.

Given the pristane-induced production of IFNα and ISGs, we examined inflammation in mice lacking the receptor for IFNα/β (IFNAR1^–/–^) and mice lacking the transcription factors, interferon response factor (IRF) 3 and IRF7, required for IFNα/β production (IRF3/7^–/–^). As expected, compared to pristane-treated WT mice, pristane-treated IRF3/7^–/–^ mice produced significantly lower levels of IFNα, whereas pristane-treated IFNAR1^–/–^ and WT mice made comparable levels (Figure 4A). Serum levels of the ISG, CXCL10, were significantly reduced in pristane-treated IRF3/7^–/–^ mice and trended to a non-significant reduction in pristane-treated IFNAR1^–/–^ mice, compared to treated WT mice (Figure 4B). Siglec-1 expression by CD11b^+^ F4/80^*int*^ inflammatory macrophages was also reduced in pristane-treated IRF3/7^–/–^ and IFNAR1^–/–^ mice, compared to WT treated mice (Figures 4C–E). In addition, levels of Mx1, ISG15, and IRF7 were significantly reduced in total peritoneal fluid cells of IFNAR1^–/–^ and IRF3/7^–/–^ treated mice, compared to WT treated controls (Figure 4F). A complete absence of IRF7 mRNA confirmed its genetic deficiency in IRF3/7^–/–^ mice.

**FIGURE 5:**
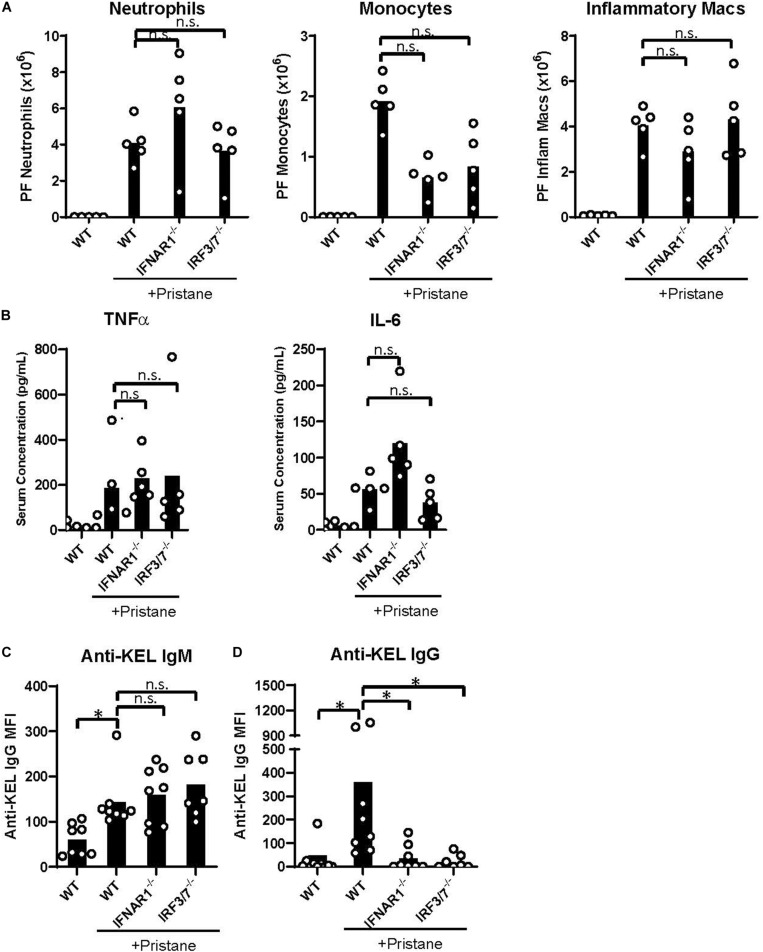
IFNα/β production and signaling promote K1 RBC alloimmunization. WT, IFNAR1^–/–^, and IRF3/7^–/–^ mice were treated with pristane 14 days prior to analysis **(A,B)** or transfusion **(C,D)**. Untreated WT mice were included as controls. **(A)** Quantification of Ly6G^+^ neutrophils, Ly6C^+^ monocytes, and CD11b^+^F4/80^*int*^ inflammatory macrophages in peritoneal fluid; gated on Zombie-negative live cells. Inflammatory macrophages were also gated on TCRβ^–^B220^–^ non-lymphocytes. **(B)** TNFα and IL-6 cytokine levels measured by multiplex array. Representative of 3 independent experiments with 5 mice per group. **(C,D)** Fourteen days after pristane-treatment, WT, IFNAR1^–/–^, and IRF3/7^–/–^ mice were transfused with K1 RBCs. **(C)** Anti-KEL IgM was measured 4–5 days after transfusion. **(D)** Anti-KEL IgG data represent the peak IgG response 21–28 days after transfusion. 7–8 mice per group. Representative of 3 independent experiments. ^∗^*p* < 0.05 by Kruskal-Wallis test with a Dunn’s post-test.

Despite the reduction in IFNα/β and ISG production in IFNAR1^–/–^ and IRF3/7^–/–^ treated mice, peritoneal infiltration by monocytes, neutrophils, and inflammatory macrophages was comparable in pristane-treated IFNAR1^–/–^, IRF3/7^–/–^ and WT mice (Figures 5A, 4C). Compared to untreated WT mice, pristane also resulted in significantly elevated levels of spleen CD11b^+^ F4/80^+^ macrophages in IFNAR1^–/–^ and IRF3/7^–/–^ mice, increased levels of spleen Ly6C^+^ monocytes and Ly6G^+^ neutrophils in IRF3/7^–/–^ mice, and trends toward increased levels in IFNAR1^–/–^ mice (Supplementary Figure 6).

Additionally, all pristane-treated groups produced comparable levels of non-IFNα/β cytokines, including TNFα and IL-6 (Figure 5B). Thus, pristane-induced peritoneal and spleen inflammation is promoted by IFNα/β-independent mechanisms.

### IFNα/β-Dependent Anti-KEL RBC Alloimmunization

Finally, given the elevated production of anti-KEL antibodies following K1 RBC transfusion, we examined the role of IFNα/β production and IFNAR1 signaling in K1 RBC alloimmunization. Following transfusion of K1 RBCs, all pristane-treated mice produced anti-KEL IgM at comparable levels (Figure 5C). However, despite the comparable levels of inflammation in all pristane-treated groups, IFNAR1^–/–^ and IRF3/7^–/–^ mice produced significantly lower anti-KEL IgG antibodies, compared to WT treated controls (Figure 5D). Thus, IFNα/β production and signaling promote K1 RBC alloimmunization in pristane-induced lupus mice.

## Discussion

Multiple studies have reported an increased frequency of RBC alloimmunization in patients with autoimmune diseases, including SLE (7–10). However, cellular and molecular mechanisms that underlie autoimmunity-induced alloimmunization have not been reported. Here, we report that IFNα/β production and signaling promote RBC alloimmunization in a lupus mouse model. To our knowledge, this is the first examination of molecular mechanisms contributing to RBC alloimmunization in a model of autoimmunity.

Two-thirds of patients with SLE express an IFNα/β gene signature that correlates with autoantibody production and disease severity. Thus, we utilized the well described pristane-induced lupus model, in which IFNα/β production and signaling have been shown to promote autoantibody production and subsequent renal pathology (41). While transient inflammation induced by viral stimuli has previously been shown to promote RBC alloimmunization (27–29), it was unclear whether chronic inflammation in autoimmune models would also induce alloimmunization. Further, in addition to IFNα, pristane induces production of numerous NFκB-induced cytokines, including IL-6, IL-1b, and TNFα, which contribute to other manifestations of lupus. For example, neutrophil invasion of the bone marrow produces TNFα, which perturbs RBC progenitor development, resulting in anemia (45). Thus, it was unclear which pristane-induced inflammatory pathways induced RBC alloimmunization.

While results reported here do not rule out a contributory role for other cytokine pathways, diminished transfusion-induced anti-KEL IgG production in pristane-treated IFNAR1^–/–^ and IRF3/7^–/–^ mice indicates a significant role for IFNα/β production and IFNAR1 signaling in K1 RBC alloimmunization. Of note, a lack of IFNα/β production or signaling did not impact markers of inflammation, including inflammatory cell infiltration in the peritoneal cavity and the spleen, nor did it impact the production of NFκB-induced inflammatory cytokines. Thus, the lack of anti-KEL IgG production in IFNAR1^–/–^ and IRF3/7^–/–^ mice was not due to reduced IFNα/β-independent inflammation.

Pristane-induced production and expansion of inflammatory cells, including neutrophils, monocytes and macrophages, likely contributed to RBC alloimmunization. These cells can phagocytose RBCs, transport antigen, and promote antigen presentation to T cells. In addition, Ly6C^+^ monocytes in the peritoneum are the primary producers of IFNα/β (48). Inflammatory macrophages in the peritoneum of pristane-treated mice expressed high levels of the ISG, Siglec-1, which was first identified as an erythrocyte binding receptor (49). It was later found to be induced by IFNα/β in patients with systemic sclerosis (47). Siglec-1^+^ macrophages are located in areas exposed to body fluids, including the peritoneum and the marginal zone of the spleen. They have been shown to capture and transfer antigen to dendritic cells and B cells for antigen presentation (50). Thus, although not tested here, Siglec-1^+^ macrophages may contribute to RBC alloantibody production.

In this transfusion model, anti-KEL IgG is clinically significant, in that it clears KEL-expressing RBCs and can induce hemolytic events, including hemolytic transfusion reactions (40). In addition to anti-KEL IgG, pristane also enhanced anti-KEL IgM production following K1 RBC transfusion. Notably, in the pristane model, enhancement of anti-KEL IgM was also present in IFNAR1^–/–^ and IRF3/7^–/–^ mice. Thus, IFNα/β is dispensable for anti-KEL IgM, but not anti-KEL IgG production. This observation indicates that IFNα/β may promote a critical process in antibody class switching. IFNα/β has been shown to promote differentiation of germinal center and antibody-producing plasma cells (51, 52). However, it also activates antigen presentation by dendritic cells, and can activate T cells directly (53). Thus, further studies are needed to determine the mechanism of IFNα/β-induced IgG class switching during RBC alloimmunization.

This report builds upon prior data implicating the IFNα/β pathway in RBC alloimmunization following acute viral infection or poly(I:C) treatment. Poly(I:C), injected 3 hr prior to transfusion, induces profoundly high transient levels of serum IFNα that diminish within 24 hr of injection (37). Consequently, K1 RBC transfusion 24 hr before or after poly(I:C) treatment does not induce anti-KEL IgG (37). In contrast, in the current study, pristane treatment resulted in chronic inflammation, IFNα production, and ISG expression that promoted RBC alloimmunization 2 to 6 weeks after pristane treatment. Pristane is thought to induce IFNα/β production by causing the infiltration of inflammatory cells that phagocytose pristane, undergo cell death, and release RNA that binds and activates TLR7 in endosomes (41). In contrast, poly(I:C) is a TLR3 agonist (54) and has been shown to bind the cytosolic receptor melanoma differentiation-associated protein 5 (MDA5), leading to activation of mitochondrial antiviral-signaling protein (MAVS) prior to IFNα/β production, which promotes RBC alloantibody production (37). Activation of disparate pathways may underly differences in quantity and chronicity of IFNα/β production. While TLR7 has been shown to be required for pristane-induced autoantibody production, other pathways warranting further investigation may promote IFNα/β-induced RBC alloimmunization.

It is noteworthy that autoantibody production induced by pristane occurs months after treatment. However, inflammation and IFNAR1 signaling within two weeks of treatment are required for later autoantibody production (41); and data presented here indicate that IFNα/β-mediated inflammation is required for RBC alloantibody production in this model. Future studies are needed to determine whether autoantibody production and autoantibody-mediated pathology promote RBC alloimmunization by IFNα/β-independent mechanisms. Such studies will have the challenge of separating the role of autoimmunity-induced alloimmunization and IFNα/β-induced alloimmunization, as autoantibody production is abrogated in IRF3/7^–/–^ and IFNAR1^–/–^ mice (41). Thus, examination of RBC alloimmunization in other lupus models with less dependence on IFNα/β may be needed.

In summary, we report that inflammation in a lupus mouse model promotes alloimmunization to the KEL RBC antigen in an IFNα/β-dependent manner. These results warrant further investigation of the role of IFNα/β in alloimmunization to other RBC antigens and of the contribution of the IFNα/β gene signature to the increased frequency of alloimmunization in patients with SLE. If these findings extend to human studies, SLE patients with an IFNα/β gene signature may benefit from personalized transfusion protocols.

## Data Availability Statement

The raw data supporting the conclusions of this article will be made available by the authors, without undue reservation.

## Ethics Statement

The animal study was reviewed and approved by the Cedars-Sinai Institutional Animal Care and Use Committee.

## Author Contributions

EM, JL, NE, CJ, MY, and DRG completed the project design and data analysis. DRG, EM, JL, NE, KN, and SP completed the experiments. MY completed the pathological analysis. DRG wrote the initial draft of the manuscript. All authors contributed to edits of the manuscript.

## Conflict of Interest

The authors declare that the research was conducted in the absence of any commercial or financial relationships that could be construed as a potential conflict of interest. The reviewer SP declared a past co-authorship with one of the authors DRG to the handling editor.
