# Supplementary material for: Type 1 Interferon Gene Signature Promotes RBC Alloimmunization in a Lupus Mouse Model
Source: Front Immunol. 2020 Sep 25;11:584254. doi: 10.3389/fimmu.2020.584254 (PMC7546415; doi:10.3389/fimmu.2020.584254)
Supplement: Supplementary file 1 [file Data_Sheet_1.PDF]

Supplementary Material

1 Supplementary Figures and Tables

1.1 Supplementary Table

Supplementary Table 1. PCR primers for quantitative real-time PCR

mRNA	Forward primer	Reverse Primer
GAPDH	5' CAT CAA GAA GGT GGT GAA GC 3'	5' CCT GTT GCT GTA GCT GTA TT 3'
Mx1	5' GAT CCG ACT TCA CTT CCA GAT GG 3'	5' CAT CTC AGT GGT AGT CAA CCC 3'
ISG15	5' GGT GTC CGT GAC TAA CTC CAT 3'	5' CTG TAC CAC TAG CAT CAC TGT G 3'
IRF7	5' TGC TGT TTG GAG ACT GGC TAT 3'	5' TCC AAG CTC CCG GCT AAG T 3'

1.2 Supplementary Figures

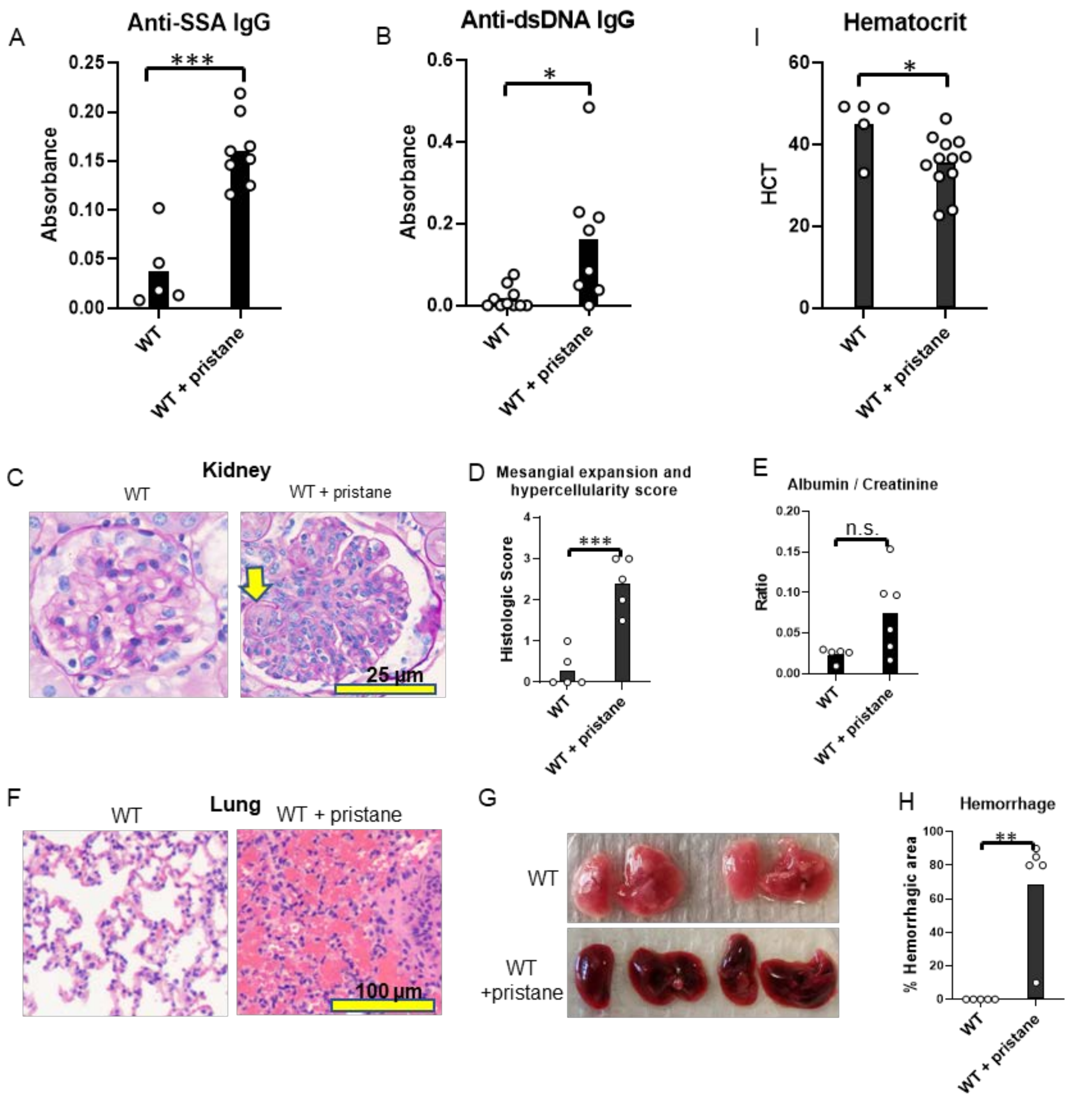

Supplementary Figure 1: Pristane induces a lupus-like phenotype. **(A,B)** Serum anti-SSA IgG and anti-dsDNA IgG autoantibodies of pristane-treated and untreated C57BL/6 mice measured by ELISA. **(C)** Histology of kidney sections from pristane-treated and untreated mice stained with periodic acid Schiff. The arrow indicates an area of endocapillary hypercellularity. **(D)** Pathologic scoring of kidney mesangial cell expansion and hypercellularity. **(E)** Urine albumin/creatinine ratio. **(F)** Histology of lung sections from pristane-treated and untreated mice stained with hematoxylin and eosin. **(G)** Photographs of representative lungs from indicated mice. **(H)** Pathologic scoring of lung hemorrhage measured by the percent of area containing RBCs. **(I)** Hematocrit of mice treated with or without pristane. Mice were treated with pristane 8 months **(A-E)** or 2 weeks **(F-I)** prior to analysis. Data are representative of 3 independent experiments. *p<0.05, **p<0.01, ***p<0.001.

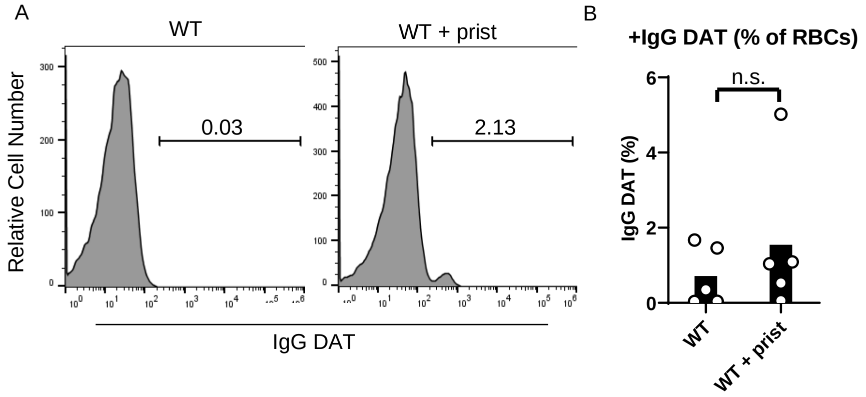

Supplementary Figure 2. Evaluation of RBC autoantibodies in pristane-treated mice. WT mice were treated with or without pristane 8 months prior to IgG direct antiglobulin testing (DAT). **(A)** Flow cytometric histograms of WT RBCs stained for anti-IgG. Numbers on flow cytometry histograms indicate percent of WT RBCs within the gated region. **(B)** Summary data showing the percent of RBCs with bound IgG. Representative of 3 independent experiments. n.s., non-significant by Mann-Whitney U test.

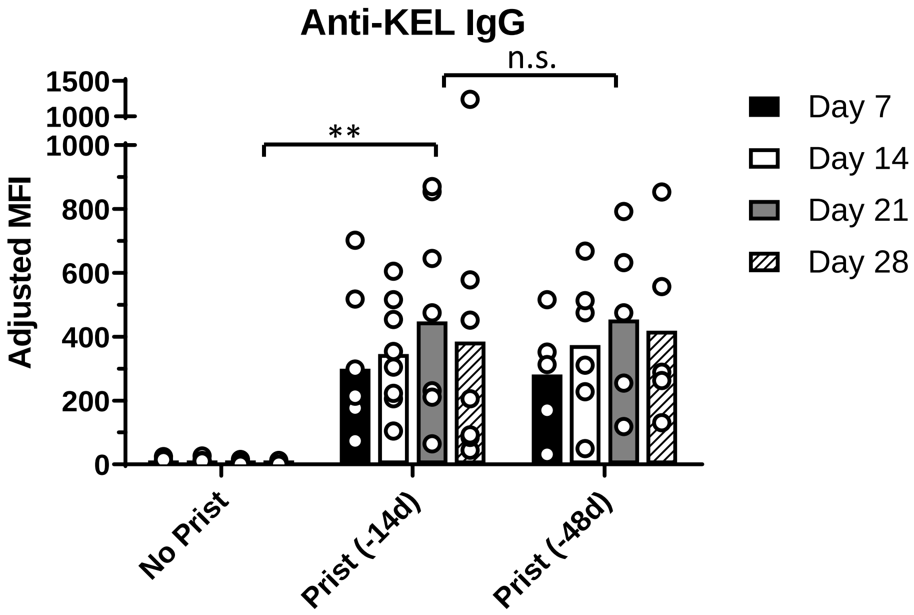

Supplementary Figure 3. Kinetics of pristane induced anti-KEL IgG alloantibodies. Recipient WT mice were transfused with K1 RBCs. Serum anti-KEL IgG in untreated mice and mice treated with pristane 14 or 48 days prior to transfusion was measured by flow cytometric crossmatch 7-28 days after transfusion. Representative of 3 independent experiments with 5-8 mice per group. Peak anti-KEL IgG levels 21 days after transfusion were compared with a Kruskal-Wallis test with a Dunn's post-test. **p<0.01, n.s. = no significant difference.

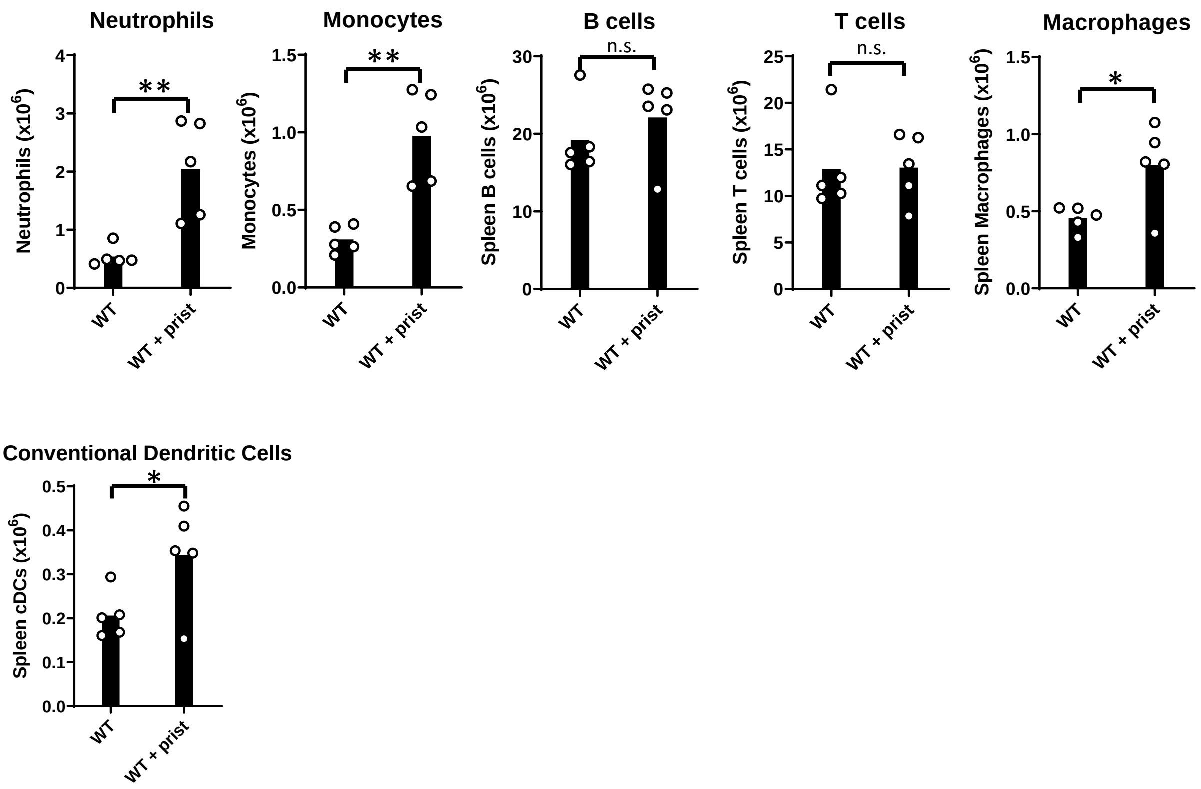

Supplementary Figure 4: Pristane-induced spleen innate immune cell expansion. WT mice were treated with or without pristane 14 days prior to flow cytometric analysis. Quantification of spleen Ly6G$^+$ neutrophils, Ly6C$^+$ monocytes, B220$^+$ B cells, TCRβ$^+$ T cells, CD11b$^+$ F4/80$^+$ macrophages, and CD11c^{hi} MHCII$^+$ conventional dendritic cells determined by flow cytometry, gated on Zombie-negative live cells. Macrophages and conventional dendritic cells were gated on TCRβ$^-$ B220$^-$ non-lymphocytes. Representative of 3 independent experiments. *$p<0.05$, **$p<0.01$, by student's t-test.

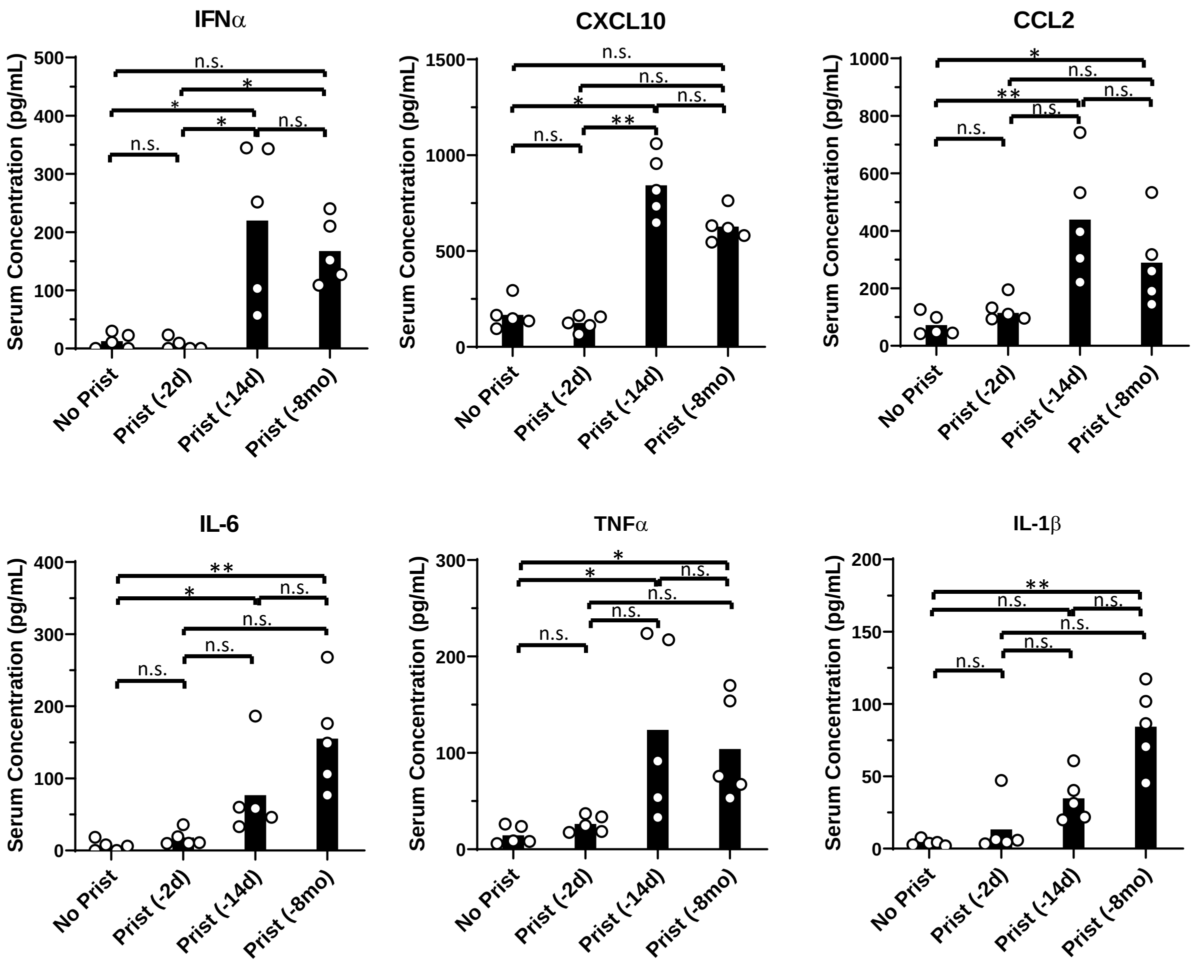

Supplementary Figure 5: Kinetics of pristane-induced cytokine production. WT mice were treated with or without pristane 2 days, 14 days, or 8 months prior to serum collection. Quantification of serum IFNα, CXCL10, CCL2, IL-6, TNFα, and IL-1β by multiplex array. Representative of 2 independent experiments. *$p<0.05$, **$p<0.01$, n.s. = no significant difference by Kruskal-Wallis test with a Dunn's post-test.

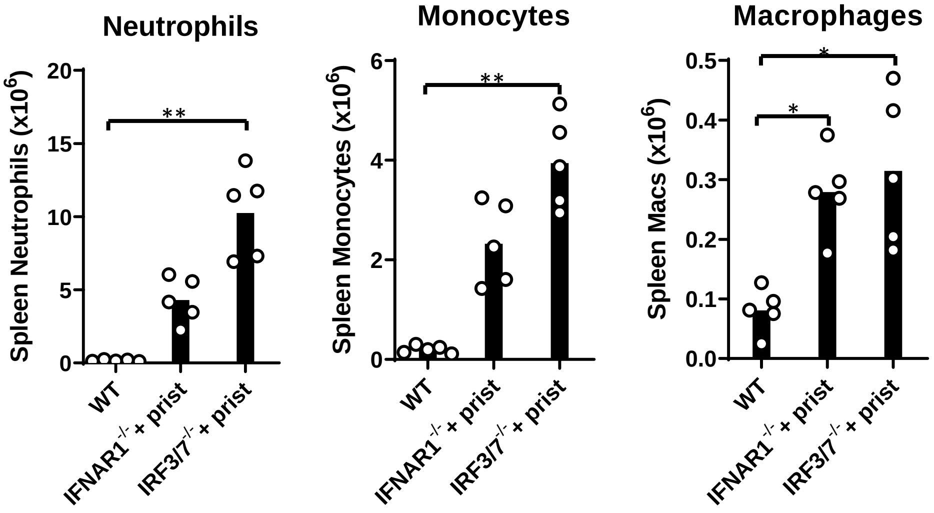

Supplementary Figure 6: IFNα/β-independent spleen inflammatory cell expansion. WT, IFNAR1$^{-/-}$, and IRF3/7$^{-/-}$ mice were treated with or without pristane 14 days prior to flow cytometric analysis. Quantification of spleen Ly6G^{+} neutrophils, Ly6C^{+} monocytes, and CD11b^{+} F4/80^{+} macrophages, determined by flow cytometry, gated on Zombie-negative live cells. Macrophages were also gated on TCRβ^{-} B220^{-} non-lymphocytes. Representative of 3 independent experiments. *$p<0.05$, **$p<0.01$, by Kruskal-Wallis test with a Dunn's post-test.
